# State-Dependent Distortions of Short-Range Internal Timing: A Narrative Review Across Stress, Anxiety, Depression, Parkinson’s Disease, and Epilepsy

**DOI:** 10.3390/jcm15020737

**Published:** 2026-01-16

**Authors:** Ekaterina Andreevna Narodova

**Affiliations:** Department of Neurology, Prof. V.F. Voyno-Yasenetsky Krasnoyarsk State Medical University, 660022 Krasnoyarsk, Russia; katya_n2001@mail.ru

**Keywords:** time perception, internal timing, stress, anxiety, depression, Parkinson’s disease, epilepsy, rhythmic behavior, biological states

## Abstract

Short-range internal timing supports coordinated movement, attention, and physiological regulation, yet distortions of time experience are frequently reported across clinical and high-arousal states. Patients with anxiety or acute stress often describe an apparent acceleration of time, whereas depressive states are more commonly associated with a slowing of subjective time. Neurological conditions, including Parkinson’s disease and epilepsy, further demonstrate alterations in temporal processing that cannot be reduced to a single mechanism. This narrative review synthesizes evidence from experimental timing paradigms, subjective passage-of-time judgments, and chronobiological approaches to examine how internal timing varies across biological states. In this study, we highlight the distinction between experiential time distortion and performance-based interval timing and discuss how task characteristics, arousal level, and neural context contribute to heterogeneous findings. Historical and methodological foundations are reviewed, including early chronobiological work linking subjective time estimation to biological rhythms. The reviewed evidence suggests that many timing distortions observed in stress-related, affective, and neurological conditions reflect state-dependent reconfiguration rather than irreversible dysfunction. Framing timing variability as a potential marker of internal state may help reconcile inconsistent results across paradigms and inform future clinical and translational research on temporal processing.

## 1. Introduction

Short-range timing supports a wide range of clinically relevant functions, including motor coordination, attention, speech, and interoceptive regulation. In everyday experience, patients often report distortions of time passage (“time flies” or “time drags”), especially under stress, anxiety, depression, and neurological disease. At the same time, experimental timing studies typically operationalize time using interval estimation, production, or reproduction tasks, which may not map directly onto subjective passage-of-time judgments. This mismatch contributes to heterogeneous findings and complicates clinical interpretation. This narrative review focuses on state-dependent distortions of internal timing across affective and neurological conditions, with an emphasis on (I) directionality (apparent speeding vs. slowing), (II) task dependence (subjective flow vs. interval timing), and (III) reversibility and context sensitivity as markers distinguishing adaptive reconfiguration from progressive dysfunction. We synthesize evidence across stress-related states, anxiety and depressive disorders, Parkinson’s disease, and epilepsy, and outline a clinically oriented interpretation of timing variability as a potential marker of state changes rather than nonspecific noise [1]. Here we argue that many timing distortions reported across affective and neurological conditions are better conceptualized as state-dependent shifts in network configuration and control stability (i.e., variability and context sensitivity), rather than as fixed, unitary timing deficits.

For this narrative synthesis, we conducted targeted searches in PubMed and Google Scholar (last accessed: 21 December 2025) using combinations of terms related to (i) passage-of-time judgments, (ii) interval timing tasks, and (iii) the clinical conditions of interest (stress/anxiety, depression, Parkinson’s disease, epilepsy). We prioritized peer-reviewed empirical studies and meta-analyses that explicitly distinguished subjective time experience from task-based timing, or that reported task parameters (interval range, paradigm, motor/working-memory load). Seminal mechanistic papers and clinically relevant reviews were included to provide conceptual continuity across conditions. Studies were emphasized when they (a) reported state manipulation or symptom-linked variability, (b) used repeated measures or reported variability indices, or (c) contrasted task types within the same sample.

Use of Generative AI and AI-Assisted Technologies (Methods): Generative AI tools were used exclusively to refine language clarity and readability; they were not used to generate scientific content, interpret data, or draw conclusions. The author reviewed and edited all AI-assisted text and takes full responsibility for the manuscript.

More broadly, developmental and environmental contexts can shape neural and cognitive signatures linked to psychopathological vulnerability, reinforcing the plausibility that ‘state’ effects on timing arise from modifiable network configurations rather than fixed deficits [2].

## 2. Historical and Methodological Foundations of Internal Time Research

Modern discussions of “internal time” in medicine emerged from at least two partially independent traditions. Chronobiology established that physiological outcomes vary systematically with circadian stage and other temporal structures. Franz Halberg’s work is central in this lineage and introduced methods aimed at quantifying temporal patterns (“chronomics”) across biological variables. Notably, Halberg and colleagues analyzed repeated self-estimations of one minute performed multiple times per day and interpreted their variation using chronobiological modeling (extended cosinor), thereby linking subjective time estimation to biological temporal organization [3,4].

In parallel, experimental psychology and cognitive neuroscience developed paradigms for interval timing (e.g., reproduction/production/bisection) and for subjective passage-of-time judgments. These approaches differ in what they measure; interval timing tasks quantify performance on defined durations, whereas passage-of-time judgments capture an experiential construct strongly modulated by arousal and affect. A clinically useful synthesis requires keeping these measurement levels distinct while examining when they converge and when they diverge across states and diseases [1].

## 3. How Distortions of Time Are Measured: Methodological Considerations

Time-related phenomena in clinical and experimental research are assessed using conceptually distinct approaches. A major source of apparent inconsistency across studies arises from the conflation of subjective passage-of-time judgments with performance-based interval timing tasks [1]. Terminology note. In this review, subjective time experience refers to the internal felt flow of time (experiential construct), whereas a passage-of-time (PoT) judgment denotes the measured report of that experience (e.g., a brief rating over a defined time window). We use ‘PoT judgment’ when referring to the assessment output and reserve ‘time experience’ for the underlying phenomenology.

Passage-of-time judgments capture an experiential construct and are typically assessed retrospectively or prospectively using verbal reports (e.g., “time passed quickly” or “time dragged”). These judgments are strongly modulated by arousal, emotional valence, and attentional engagement, making them particularly sensitive to stress and affective states. In contrast, interval timing tasks—such as time production, reproduction, or bisection—require participants to estimate or generate specific durations, often in the range of hundreds of milliseconds to several seconds. Performance in these tasks depends on attentional resources, working memory, and motor execution, and may dissociate from subjective experience. For example, individuals reporting accelerated time passage under anxiety may simultaneously show overestimation or underestimation of intervals depending on task demands and threat context [1,3].

Chronobiological approaches provide a complementary perspective by examining temporal estimation repeatedly across biological cycles. Early work in chronobiology demonstrated that self-estimation of fixed durations (e.g., one minute) varies systematically with biological rhythms, suggesting that internal time is embedded within broader temporal organization rather than operating as an isolated cognitive function. Recognizing these methodological distinctions is essential for interpreting timing distortions across clinical populations. Apparent contradictions in the literature often reflect differences in what aspect of “time” is being measured, rather than true disagreement about underlying processes.

Several key task parameters were used to investigate why objective timing tasks yield heterogeneous results. In addition to construct mismatch (PoT vs. interval timing), heterogeneity across interval timing findings likely reflects systematic differences in task parameters: (i) interval range (sub-second vs. supra-second) with different reliance on motor/predictive vs. working-memory/executive resources; (ii) paradigm (production/reproduction vs. discrimination/bisection), which differentially loads motor output and memory; (iii) prospective vs. retrospective instructions and attentional allocation; (iv) motor and cognitive load, including concurrent tasks and response modality; and (v) whether studies report precision/variability metrics (trial-to-trial SD/CV) rather than only mean bias. These parameters can drive opposite directional effects even within the same condition, whereas variability-related outcomes may show greater cross-study consistency.

An overview of state-dependent timing alterations across conditions, task types, and typical directions is provided in Table 1.

## 4. Stress and High-Arousal States

### 4.1. Acute Experimental Stress (Lab Stressors)

Acute stress and high arousal reliably influence subjective time perception, but the direction and magnitude of effects depend on both task type and operationalization of “time”. A key distinction is between subjective passage-of-time judgments and objective interval timing. In high-arousal contexts (e.g., threat, fear), many individuals retrospectively report that “time seemed to speed up” or “moments felt brief,” consistent with a heightened vigilance state and increased sympathetic activation. For example, in experimental settings involving threat or physiological stressors, participants often report alterations in the subjective flow of time even when interval timing tasks (e.g., reproduction or production) do not show consistent directional shifts. This dissociation suggests that subjective changes in time passage under stress may relate more to affective salience and memory encoding than to a single scalar timing mechanism [1,3].

In contrast, objective timing tasks under acute stress have yielded heterogeneous results. Some studies report overestimation of intervals under stress, whereas others find underestimation or null effects depending on paradigm demands (e.g., prospective vs. retrospective timing), suggesting that task constraints (attention, working memory) modulate how stress impacts temporal performance.

### 4.2. Acute Stress in Non-Clinical Samples

Experimental work in healthy participants indicates that acute arousal can influence judgments of time passage, although effects vary with task design and context [3]. For example, participants exposed to acute psychosocial stressors often judge intervals as shorter when recollecting after the fact, aligning with subjective reports of “time flying” during intense experiences.

### 4.3. Everyday High-Demand Contexts (Ecological)

Everyday high-demand contexts (e.g., performance pressure) are often accompanied by strong subjective changes in time experience. However, direct experimental evidence is heterogeneous and strongly depends on whether studies assess passage-of-time judgments or interval timing under controlled conditions [1,3].

## 5. Anxiety Disorders

Anxiety disorders are characterized by heightened vigilance, anticipatory fear, and amplified responses to threat—features that systematically influence temporal perception [3,7,8]. We distinguish between transient threat-induced anxiety (experimental manipulations), trait anxiety dimensions, and clinical anxiety disorders, as these levels can differentially modulate attentional control, interoceptive salience, and task performance.

### 5.1. Subjective Time in Anxiety

Subjective reports from individuals with generalized anxiety disorder (GAD) frequently indicate that “time feels sped up” or “fleeting,” particularly in contexts of uncertainty. Studies using retrospective judgment tasks demonstrate that individuals with clinically significant anxiety are more likely than controls to report accelerated passage of time, consistent with arousal–attentional accounts [8].

### 5.2. Interval Timing in Anxiety

When anxiety is probed using interval timing tasks (e.g., time estimation, reproduction), results are more nuanced. For instance, under threat of shock, participants often overestimate intervals, possibly reflecting increased pacemaker rate under high arousal. However, this effect is sensitive to task demands; when cognitive resources are heavily taxed, anxious participants may show underestimation due to attentional distraction [1,7,8].

Together, these findings suggest that anxiety alters multiple components of temporal processing—from pacemaker rate to attentional allocation—and that apparent “acceleration of time” in anxiety cannot be reduced to a single mechanism.

## 6. Depression

Depressive disorders are frequently associated with slowed subjective time and alterations in temporal processing across tasks.

### 6.1. Passage-of-Time Judgments in Depression

Patients with major depressive disorder (MDD) commonly describe time as “dragging” or “slow,” particularly in periods of low positive affect and social withdrawal. Several clinical and experimental studies report that depressive symptom severity correlates with slower subjective time passage, especially in self-report measures where individuals reflect on the flow of time over extended periods [5,6].

### 6.2. Interval Timing in Depression

Experimental studies using interval timing tasks (e.g., reproduction, production) in depressed populations demonstrate mixed findings. Some work indicates that depressed individuals overestimate intervals, interpreted as a slowed internal clock, while others find no significant group differences or context-dependent effects influenced by attentional engagement and task difficulty [1,5]. These mixed results may arise from heterogeneity in depressive presentations and from the fact that interval timing tasks capture neurocognitive components distinct from subjective passage-of-time experiences [6,8]. Heterogeneity in depression-related interval timing likely reflects moderating factors beyond diagnosis status alone. Symptom severity, psychomotor slowing, and cognitive control demands may differentially affect production/reproduction paradigms (motor output and working memory) versus perceptual discrimination tasks. Depressive subtypes (e.g., melancholic features with prominent psychomotor retardation versus anxious depression) and medication status may further contribute to divergent results, suggesting that variability itself may be a more robust signal than a uniform directional shift.

## 7. Parkinson’s Disease and Dopaminergic Timing

### 7.1. Core Features of Temporal Disturbances in Parkinson’s Disease

Parkinson’s disease (PD) is a neurodegenerative disorder characterized by progressive dysfunction of dopaminergic pathways, particularly within the nigrostriatal system. Beyond its well-known motor manifestations, PD has been repeatedly associated with disturbances in temporal processing, especially in tasks requiring the estimation, production, or reproduction of time intervals. These temporal alterations have been widely interpreted as reflecting changes in internal timing mechanisms linked to dopaminergic modulation [9,10,11].

Experimental studies indicate that patients with PD show impaired performance in interval timing tasks across a range of durations, most prominently in the seconds range. Compared with healthy controls, individuals with PD often demonstrate reduced temporal precision and increased variability, suggesting a disruption in the stability of internal timing rather than a uniform directional bias [1,9,10,11].

### 7.2. Interval Timing Performance in Parkinson’s Disease

Multiple experimental studies using interval timing paradigms—including time production, reproduction, and temporal bisection—have documented characteristic timing deficits in PD, such as increased variability and reduced temporal precision [9]. Classic work by Harrington and colleagues demonstrated that patients with PD tend to reproduce intervals as longer than intended, consistent with a slowing or miscalibration of internal timing processes. Subsequent studies have replicated these findings across different task designs and temporal ranges, reinforcing the notion that dopaminergic dysfunction affects the regulation of internal temporal representations rather than isolated perceptual judgments [11].

Importantly, these impairments are not uniform across all patients or tasks. Performance varies with disease severity, cognitive status, and task demands, indicating that temporal dysfunction in PD reflects an interaction between neurochemical deficits and higher-order cognitive control.

### 7.3. Subjective Experience of Time in Parkinson’s Disease

In addition to task-based measures, patients with PD frequently report alterations in the subjective experience of time. Qualitative descriptions include a sense that time passes more slowly, feels fragmented, or lacks continuity, particularly during periods of motor slowing or cognitive fatigue. These experiential reports do not always align directly with performance on interval timing tasks, highlighting a dissociation between subjective passage-of-time judgments and objective temporal estimation [1].

This dissociation is clinically relevant, as it suggests that timing disturbances in PD are not limited to a single cognitive mechanism but involve broader changes in how temporal information is integrated with motor and affective states. PD therefore represents a valuable clinical model for examining how neurobiological alterations translate into both measurable timing deficits and altered temporal experience.

### 7.4. Dopaminergic Modulation and Pharmacological Effects

The central role of dopamine in temporal processing is supported by both experimental and pharmacological evidence. Dopaminergic depletion is widely considered a key contributor to timing disturbances in PD, and dopaminergic treatment has been associated with partial improvements in temporal accuracy and/or variability in some paradigms [9,10,11].

Clinical studies examining the effects of dopaminergic therapy provide partial support for this framework. Administration of levodopa or dopamine agonists has been reported to improve temporal accuracy and reduce variability in some interval timing tasks, although effects are not universal and depend on task structure and individual patient characteristics. These findings suggest that dopamine modulates the stability and calibration of internal timing rather than exerting a simple linear effect on perceived duration [10].

### 7.5. Heterogeneity and Moderating Factors

Temporal disturbances in PD exhibit substantial interindividual variability. Factors influencing timing performance include disease stage, cognitive impairment, comorbid affective symptoms, and pharmacological treatment status. Patients with greater executive dysfunction or depressive symptoms often show more pronounced timing variability, underscoring the contribution of non-motor features to temporal processing deficits. Taken together, evidence from PD supports the view that internal timing is a state-dependent function influenced by neurochemical balance, cognitive resources, and behavioral context. Rather than reflecting a fixed deficit, temporal disturbances in PD appear to arise from reduced stability of timing control, making this condition particularly informative for understanding how biological states shape temporal organization.

Phenotype-level differences (e.g., tremor-dominant vs. akinetic-rigid presentations) may also contribute to variability in timing outcomes through differential motor and executive burdens, although the current timing literature does not yet provide consistent phenotype-stratified evidence; future studies should report phenotype and motor subtype when feasible.

Although dopaminergic modulation is central, timing variability in PD likely also reflects broader network-level changes involving fronto-striatal executive control, cerebellar–cortical contributions to temporal prediction, and non-dopaminergic systems that shape arousal and cognitive stability. This broader view aligns with the present framework by emphasizing stability/variability of temporal control as an emergent property of distributed networks rather than a single neurotransmitter-driven ‘clock’.

## 8. Epilepsy and Temporal Disturbances

### 8.1. Alterations of Temporal Processing in Epilepsy

Epilepsy is increasingly recognized as a disorder extending beyond paroxysmal seizures, involving persistent alterations in cognition, perception, and subjective experience. Temporal processing represents one such domain, where both objective timing performance and subjective experience of time may be altered even during interictal periods. These disturbances are heterogeneous and depend on epilepsy type, seizure focus, comorbidities, and treatment status [12,13,14].

Experimental studies investigating interval timing in epilepsy have reported deficits in temporal estimation, reproduction, and discrimination, particularly in tasks involving seconds-range intervals. Compared with healthy controls, patients with epilepsy often demonstrate increased variability and reduced consistency in timing performance, suggesting impaired temporal stability rather than a uniform directional bias toward acceleration or slowing [14,15].

### 8.2. Interval Timing and Behavioral Variability in Epilepsy

Interval timing paradigms provide evidence that epilepsy is associated with disrupted temporal precision. Studies in focal epilepsy, especially temporal and frontal lobe epilepsy, indicate that patients may show altered reproduction of time intervals and increased trial-to-trial variability. These effects are often independent of seizure frequency and may persist despite clinical seizure control, pointing to broader network-level alterations. Notably, timing variability in epilepsy is not necessarily constant. Fluctuations in performance have been observed across testing sessions, suggesting sensitivity to internal state factors such as fatigue, attention, medication effects, and subclinical epileptiform activity. This pattern aligns with a state-dependent interpretation of temporal disturbances rather than a fixed deficit model.

Seizure focus and timing phenotype (conceptual contrast). Timing disturbances may differ by seizure focus. Temporal lobe epilepsy, particularly mesial temporal involvement, is more likely to be associated with phenomenological alterations of experience (including subjective continuity and passage-of-time distortions) and memory-linked contextual effects, whereas frontal lobe epilepsy may more strongly impact executive control and attentional stability, which can disproportionately affect task-based interval timing performance and trial-to-trial variability. While direct head-to-head comparisons remain limited, existing evidence that lateralized temporal foci can modulate interval timing performance supports the broader interpretation that timing outcomes depend on which networks are destabilized (experiential/interoceptive vs. executive/motor control).

### 8.3. Subjective Experience of Time in Epilepsy

In addition to task-based findings, patients with epilepsy frequently report alterations in subjective time experience. Descriptions include episodes of accelerated or slowed time perception, temporal fragmentation, and altered continuity of experience. Such reports may occur in association with seizures, during auras, or interictally, particularly in individuals with temporal lobe involvement [12,13].

Importantly, subjective distortions of time in epilepsy do not always correspond directly to performance on interval timing tasks. This dissociation mirrors observations in affective disorders and Parkinson’s disease, reinforcing the distinction between experiential time and experimentally measured temporal intervals. In clinical contexts, subjective time alterations may therefore provide complementary information about internal state and network instability that is not captured by standardized tasks alone [9,10,14,15].

For clarity, epilepsy-related temporal distortions can be considered across three clinically distinct contexts: (i) interictal timing variability (baseline network instability and cognitive state factors), (ii) peri-ictal changes (pre-/postictal fluctuations linked to transient network reconfiguration), and (iii) ictal or aura-related distortions (phenomenological alterations during seizures). These contexts likely involve different mechanisms and have different implications for the interpretation of subjective reports versus task performance.

### 8.4. Epilepsy as a Model of State-Dependent Timing Instability

Epilepsy offers a unique clinical model for examining state-dependent temporal instability. Fluctuations in arousal, network excitability, and cognitive control are intrinsic features of the disorder and may modulate internal timing even outside overt seizures. From this perspective, temporal disturbances in epilepsy may reflect transient reconfigurations of neural timing networks rather than permanent dysfunction [12,14].

Recent narrative syntheses have emphasized the relevance of behavioral rhythms, self-monitoring, and temporal structure in epilepsy management, highlighting how variations in daily patterns can inform clinical assessment and self-management strategies. Within this broader framework, alterations in internal timing may be understood as part of a dynamic interaction between neural excitability, cognitive state, and environmental demands rather than as isolated impairments [14,15,16].

### 8.5. Clinical and Conceptual Implications

The evidence reviewed suggests that temporal disturbances in epilepsy are multifaceted and context-sensitive. Increased variability, dissociation between subjective and objective timing, and sensitivity to internal state changes are recurrent features across studies. These characteristics support the interpretation of altered timing in epilepsy as a marker of network instability rather than as a simple consequence of seizure activity [14]. From a clinical standpoint, attention to temporal experience and timing variability may complement conventional assessments focused on seizure frequency and severity. Although current evidence does not support the use of timing measures as diagnostic tools, they may offer insight into cognitive and experiential aspects of epilepsy that are relevant for patient-centered care and future research [13,15].

## 9. Integrative Synthesis: State-Dependent Variability of Internal Timing Across Conditions

Across stress-related states, affective disorders, Parkinson’s disease, and epilepsy, a convergent pattern emerges despite substantial heterogeneity in methods and outcomes. Alterations of internal timing rarely present as uniform acceleration or slowing. Instead, the literature consistently points to increased variability, context sensitivity, and dissociation between subjective and objective temporal measures as shared features across conditions [11,12,13,14].

One of the most robust observations across domains is the dissociation between subjective passage-of-time experience and performance in interval timing tasks. Stress and anxiety are frequently associated with a subjective sense of accelerated time, whereas depression is more commonly linked to a slowing of experienced time. In contrast, interval timing tasks reveal mixed and task-dependent effects, often characterized by increased variability rather than a consistent directional bias. Similar dissociations are evident in Parkinson’s disease and epilepsy, where subjective temporal experience and task-based timing performance may diverge substantially. This dissociation suggests that experiential and operational aspects of time rely on partially overlapping but distinct mechanisms and should not be interpreted interchangeably in clinical research [12,13,14].

**Figure 1:**
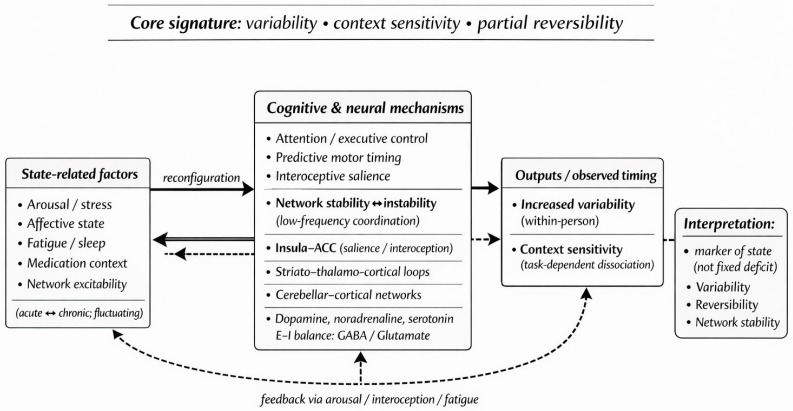
State-dependent variability framework for internal timing distortions.

A schematic summary of the relationships between state factors, timing mechanisms, and observed temporal variability is shown in Figure 1.

To provide anatomical specificity, the proposed stability framework can be anchored to recurrent timing-related substrates, including insula–anterior cingulate (salience/interoceptive monitoring; particularly relevant to stress/anxiety), striato-thalamo-cortical loops supporting seconds-range interval timing (relevant to PD), and cerebellar–cortical networks contributing to temporal prediction and calibration under uncertainty. This view is consistent with broader evidence that large-scale networks can reorganize dynamically via compensatory pathways (e.g., state-dependent interhemispheric interactions supporting recovery after focal lesions) [17].

State-related factors (arousal/stress, affective state, neuromodulatory context, network excitability, fatigue, and medication status) modulate distributed timing-control mechanisms, including attentional and executive control, predictive motor timing, interoceptive salience, and low-frequency network coordination. At the neurobiological level, these processes involve interactions across salience-related regions (e.g., insula–anterior cingulate cortex), striato-thalamo-cortical loops supporting seconds-range interval timing, and cerebellar–cortical networks contributing to temporal prediction and calibration. Neuromodulatory influences extend beyond dopamine to include noradrenergic and serotonergic systems, as well as excitation–inhibition balance (GABA/glutamate), which together shape the stability of temporal control.

Across conditions, the most reproducible signature is characterized by (i) increased within-person variability, (ii) context sensitivity with dissociation between passage-of-time judgments and interval timing performance, and (iii) partial reversibility when state factors normalize, reflecting shifts in network stability rather than a unitary fixed deficit. Bidirectional feedback loops indicate that altered timing output can, in turn, influence arousal, interoceptive awareness, and fatigue, reinforcing state-dependent dynamics. Specifically, the feedback arrow denotes a secondary response whereby perceived timing instability (or loss of temporal control) may elicit increased vigilance and stress/arousal, which can further modulate timing mechanisms in a self-reinforcing loop, thereby creating a self-reinforcing arousal–timing loop.

Timing disturbances fluctuate with arousal, cognitive load, fatigue, medication status, and momentary network conditions. In Parkinson’s disease, dopaminergic state and cognitive capacity modulate temporal precision; in epilepsy, timing variability may change across interictal states independent of seizure frequency. These observations argue against a fixed-deficit model and instead support the view that internal timing reflects the current functional configuration of distributed neural systems [1]. From a systems perspective, timing disturbances across conditions can be understood in terms of reduced stability of temporal organization. Rather than invoking a single internal clock mechanism, the reviewed evidence favors a model in which internal timing emerges from interactions among attentional control, predictive motor processes, interoceptive signals, and low-frequency neural dynamics. When these interactions are well coordinated, timing behavior remains relatively stable. When coordination weakens—due to stress, affective disturbance, neurochemical imbalance, or network pathology—temporal output becomes more variable and less predictable.

Conceptual frameworks emphasizing rhythmic coordination and low-frequency network stability provide a useful background for interpreting these findings. In epilepsy research, for example, narrative syntheses have highlighted how behavioral rhythms and self-monitoring reflect broader network dynamics rather than isolated symptoms. Similarly, mechanistic perspectives on rhythmic entrainment and network stability underscore the role of low-frequency organization in shaping temporal behavior across states. Within this context, timing variability may be viewed not as noise but as a marker of transient instability in temporal control, with potential relevance across neurological and psychiatric conditions [18,19,20].

Traditional internal clock models (e.g., pacemaker–accumulator accounts) provide a useful baseline for interpreting directional biases in interval timing under arousal or dopamine manipulation. However, the present synthesis emphasizes that across clinical conditions, the most reproducible signature is often not a uniform speeding or slowing, but increased variability, context sensitivity, and dissociation between experiential and task-based measures. This shifts the interpretive focus from a single rate parameter to the stability of distributed timing control under changing biological states.

Importantly, reversibility is a recurring theme. Timing disturbances often attenuate when internal or external conditions normalize, such as reductions in stress, effective pharmacological treatment, or restoration of cognitive control. This reversibility distinguishes state-dependent timing alterations from progressive deterioration and supports their interpretation as adaptive or compensatory reconfigurations rather than purely pathological deficits. Taken together, the evidence synthesized in this review supports a unified interpretation of temporal disturbances across diverse conditions; internal timing is a state-sensitive, dynamically organized function, whose variability reflects changes in biological and cognitive context. Recognizing this common structure may help reconcile inconsistent findings across paradigms and guide future research toward more nuanced, state-aware approaches to temporal processing in clinical populations.

State–trait continuum and limits of reversibility. We clarify that “state-dependent” does not imply that all timing alterations are fully reversible. Rather, timing behavior may reflect a state–trait continuum, where transient fluctuations (stress load, sleep/fatigue, medication state, arousal) operate on top of slower structural or neuroplastic changes. In progressive neurodegenerative disease, baseline network degradation can reduce the ceiling for recovery, making reversibility partial or context-bound (e.g., improvement with dopaminergic state or cognitive support without full normalization). In major depression, chronic symptom burden and neuroplastic adaptations may similarly shift baseline timing stability, while still allowing short-term state-linked fluctuations. Within this framework, “reversibility” is best interpreted as within-person modulation and potential attenuation when state drivers normalize, not as a guarantee of complete restoration in advanced disease.

## 10. Clinical Implications and Future Directions

The synthesis presented in this review has several implications for clinical research and practice. First, disturbances of internal timing should not be interpreted solely as fixed cognitive deficits. Across stress-related states, affective disorders, Parkinson’s disease, and epilepsy, timing alterations frequently exhibit state dependence and reversibility, suggesting sensitivity to internal and contextual factors rather than irreversible dysfunction [9,11].

Second, the distinction between subjective passage-of-time experience and task-based interval timing performance is clinically relevant. Subjective reports of time acceleration or slowing may provide information about affective state, arousal, or network instability that is not captured by standardized timing tasks. Conversely, increased variability in interval timing performance may reflect reduced stability of temporal control even in the absence of overt subjective complaints. Integrating both experiential and behavioral measures may therefore offer a more comprehensive view of temporal disturbances in clinical populations [12,14].

Third, timing variability may serve as a transdiagnostic marker of altered internal state rather than a disorder-specific signature. Similar patterns of increased variability and context sensitivity appear across neurological and psychiatric conditions, raising the possibility that temporal measures could complement existing phenotyping approaches. However, current evidence does not support their use as standalone diagnostic tools.

### Practical Assessment Options and Testable Study Designs

To increase clinical and translational utility, we propose practical options that separate experiential time/PoT judgments (brief ratings of felt time) from interval timing task performance, and that prioritize within-person variability and context sensitivity as clinically informative readouts. Here, PoT judgment refers to a brief subjective report of time experience over a defined window (e.g., VAS/short item), rather than performance on an interval timing task.

(A) Low-burden candidate assessments include (i) a single-item PoT rating (PoT judgment) (e.g., “time feels faster/slower than usual”) recorded repeatedly across contexts (e.g., high stress vs. rest; morning vs. evening) to capture fluctuations; and (ii) a brief interval timing task (e.g., short reproduction or production trials) with repeated trials to estimate within-person variability (SD/CV) and dissociation indices between PoT judgment and task performance.
(B) Testable designs include (i) within-subject state manipulation (baseline → stress/threat induction → recovery) to examine reversibility and context sensitivity; (ii) longitudinal symptom-linked monitoring across days/weeks paired with sleep/fatigue and symptom severity scales to test whether variability tracks internal state; and (iii) task-parameter probing (sub-second vs. supra-second; discrimination vs. reproduction) to explain heterogeneous results and identify paradigms most sensitive to state changes.
(C) Illustrative workflow: In epilepsy or depression follow-up, a patient could complete a PoT rating plus a one-minute timing task alongside routine PROs (sleep, fatigue, mood). Increased variability and marked PoT–task dissociation would be interpreted as potential indicators of reduced timing-control stability, prompting targeted review of state factors (recent stress load, sleep disruption, medication changes) rather than being treated as a fixed cognitive deficit.

For future research, several directions appear particularly important. Longitudinal designs are needed to examine how timing disturbances fluctuate with changes in symptoms, treatment, and daily context. Studies should explicitly report task characteristics and distinguish between different temporal constructs to improve comparability across investigations. Finally, incorporating continuous or repeated measures of timing may help capture dynamic state changes that are missed by single-session assessments.

## 11. Limitations

This review has several limitations that should be acknowledged. First, as a narrative review, it does not provide a quantitative synthesis of effect sizes, and the strength of evidence varies across conditions and paradigms. Although the literature on temporal processing is extensive, methodological heterogeneity limits direct comparison between studies. Second, many investigations rely on small sample sizes and cross-sectional designs, reducing generalizability and limiting causal inference. Differences in task design, interval range, and instructions further contribute to inconsistent findings, particularly when subjective and objective measures of time are conflated. Third, subjective reports of time experience are inherently influenced by memory, language, and introspective ability, which may be altered in clinical populations. As a result, subjective passage-of-time judgments should be interpreted cautiously and in conjunction with behavioral data.

Medication effects represent an important confound in clinical timing studies. Antidepressants and anxiolytics may alter arousal and attentional control; antiepileptic drugs may affect processing speed, vigilance, and variability; dopaminergic therapy in PD can change both motor output and timing calibration. Many studies do not report medication status in sufficient detail, which limits interpretability and may partly explain heterogeneous results across tasks and samples. Importantly, medication influences may reflect both acute dose-dependent effects on timing and arousal and longer-term neuroadaptive changes with chronic treatment, which may contribute differently to state-like versus trait-like timing alterations.

Finally, the review focuses on short-range internal timing and does not address longer temporal scales, such as circadian rhythms, in detail. Interactions between short-range timing and longer biological rhythms may be relevant but fall outside the scope of the present synthesis.

## 12. Conclusions

Distortions of internal timing are a common but often underappreciated feature across stress-related states, affective disorders, and neurological conditions. Evidence reviewed here suggests that these disturbances are best understood as state-dependent and dynamically organized phenomena, characterized by increased variability, context sensitivity, and dissociation between subjective experience and task-based performance. Rather than reflecting a single impaired mechanism, altered timing appears to emerge from changes in the coordination of cognitive, affective, and neurobiological processes. Recognizing internal timing as a flexible, state-sensitive function may help reconcile heterogeneous findings across paradigms and conditions. Future research adopting state-aware and methodologically transparent approaches may clarify the clinical relevance of temporal measures and their potential role in understanding adaptive and maladaptive changes in brain–behavior dynamics.

## Figures and Tables

**Table 1:** Overview of State-Dependent Distortions of Internal Timing across Conditions.

Condition	Timing Measure	Typical Pattern	Task Paradigm	Key References
Acute stress/high arousal	Passage-of-time	Faster	Retrospective verbal judgment	[1,2,5,6]
Acute stress/high arousal	Interval timing	Mixed	Time reproduction/production	[1,2]
Anxiety disorders	Passage-of-time	Faster	Retrospective judgment	[6,7]
Anxiety disorders	Interval timing	Faster or mixed	Time reproduction/production	[1,6,7]
Depression	Passage-of-time	Slower	Retrospective self-report	[4,5]
Depression	Interval timing	Mixed	Time reproduction/production	[1,4,7]
Parkinson’s disease	Interval timing	Slower/increased variability	Reproduction, bisection	[8,9,10]
Parkinson’s disease	Passage-of-time	Slower/fragmented	Qualitative reports	[1,8]
Epilepsy (focal/temporal)	Interval timing	Increased variability	Reproduction, discrimination	[11,12,13,14]
Epilepsy (temporal lobe)	Passage-of-time	Faster/slower/fragmented	Subjective reports, aura-related	[11,12,15]

‘Typical pattern’ indicates the direction most consistently reported across the referenced studies for a given measure (passage-of-time vs. interval timing) and condition. When findings were inconsistent across paradigms, interval ranges, or task demands, we labeled the pattern as ‘mixed’ and emphasized variability/stability outcomes rather than a single directional bias. This table is intended as a qualitative orientation tool rather than a quantitative summary.

## Data Availability

No new data were created or analyzed in this study. Data sharing is not applicable to this article.
